# Geospatial truck parking locations data for Europe

**DOI:** 10.1016/j.dib.2024.110277

**Published:** 2024-03-01

**Authors:** Steffen Link, Patrick Plötz

**Affiliations:** a Fraunhofer Institute for Systems and Innovation Research ISI, Breslauer Strasse 48, 76139 Karlsruhe, Germany; b Karlsruhe Institute of Technology (KIT), Institute of Electrical Engineering (ETI), Kaiserstraße 12, 76131 Karlsruhe, Germany

**Keywords:** GPS coordinates, Trucking parking facilities, TEN-T, Heavy-duty trucks, Charging infrastructure, Mean-shift clustering

## Abstract

This data article introduces a comprehensive dataset of real-world truck parking locations across Europe. The dataset comprises *N* = 19,713 designated parking sites classified according to public accessibility and suitability for heavy-duty trucks (HDTs). More specifically, core information comprises the truck stop category, latitude and longitude information, area size, and country assignment. Furthermore, additional information such as truck traffic flow volumes, proximity to the highway network, and land use information provide supplemental data on ambient conditions and thus enhance the contextual relevance of those locations.

The dataset was systematically generated using OpenStreetMap (OSM) data, focusing on parking areas, rest areas, and fueling stations as predominant public truck parking sites. These locations were evaluated and filtered for truck accessibility and suitability and then complemented and validated using commercial truck routing / geocoding software. Further refinement was achieved by Mean-Shift clustering. The further integration of supplementary datasets increased the information level, and all clustered locations were labeled into four archetypal categories. Finally, filtering retained only confidently classified publicly accessible and truck-certified parking and service facilities.

This dataset assists in finding real-world stop options for HDTs during national or international operations and identifying suitable and most attractive sites for deploying alternative charging or refueling infrastructures along the European transport network. Accordingly, it can serve as a valuable resource for research in traffic science, future energy systems, and alternative truck powertrains. Its added value extends to diverse stakeholders like Charge Point Operators (CPOs), truck manufacturers, logistics companies, and public authorities.

Specifications Table

**Table:** 

Subject	Business, Management and Decision Sciences. Transportation Management
Specific subject area	Transportation management; Truck route planning; Future road freight infrastructure planning
Data format	Filtered Analyzed Processed (feature and additional information)
Type of data	Table (.csv)
Data collection	We systematically explored OpenStreetMap data to identify public parking locations that are accessible and certified for trucks, encompassing parking areas, rest areas, and fueling stations. We utilized further data from commercial truck routing software to validate and complement these findings. Employing the Mean-Shift clustering algorithm, we merged and clustered the geo-coordinates of nearby locations to condense the dataset. Finally, refining and enhancing the dataset involved supplementary datasets and several filters. The final dataset solely contains those locations meeting the criteria of being publicly accessible and truck-certified with a certain confidence.
Data source location	OpenStreetMap [1] via Overpass API [2] Commercial truck routing software: [3], [4], [5] Ten-T road network information: [6] Corine Land Cover 2018: [7] Truck traffic flow data: [8]
Data accessibility	Repository name: Zenodo Data identification number: v03 URL to data: https://zenodo.org/doi/10.5281/zenodo.10077459 Instructions for accessing these data: Apart from the actual truck parking location dataset in .csv format, this repository provides an interactive map (.html), an additional codebook (.csv), and a brief documentation (.pdf).

## 1 Value of the Data

• This dataset contains *N* = 19,713 real-world truck parking locations. It assists in finding real-world stop options for HDTs during national or international operations. Accordingly, it represents a crucial resource for identifying suitable and most attractive sites for deploying alternative infrastructure along the European transport network, comprising charging stations for battery-electric trucks (BETs) and hydrogen refueling stations (HRS) for fuel-cell electric trucks (FCETs).
• Traffic and transport scientists may integrate those locations as candidates when planning alternative infrastructure networks for electrified HDT fleets or as references in vehicle-routing problems when optimizing current or future operations. Different optimization models, such as node-based, tour-based, or traffic-flow/path-based approaches, agent-based modeling, and heuristics may aid in selecting optimal or most attractive locations and best-fit tour routings.
• Energy system scientists may use those locations to predict regionalized energy demand from alternative infrastructures, particularly charging locations.
• Industry stakeholders like CPOs or truck manufacturers may utilize this dataset to identify potential locations and initiate alternative infrastructure sites where trucks already stop today, easing the market diffusion of low-carbon trucks and realizing new business potential. Truck engineers may anticipate the implications of different network densities and infrastructure availability on the sizing of on-board storage systems, particularly batteries or hydrogen tanks. When designing their grid network or scheduling potential expansions, grid network operators may anticipate the future energy demand at such locations. Logistic companies may utilize this dataset to identify stop options for their operating HDT fleets.
• Public authorities may utilize these identified locations as potential candidates, aligning their efforts with the European Alternative Fuels Infrastructure Regulation (AFIR) and speeding up alternative infrastructure development along the Trans-European Transport Network (TEN-T).

## 2 Background

The fast electrification of HDTs is pivotal in limiting global warming in line with the Paris Climate Agreement [9,10], but insufficient alternative infrastructure deployments are widely perceived as a major barrier [11], [12], [13]. Ideally and presumably most effectively, existing truck parking locations may be equipped and upgraded with such alternative infrastructure, thus seamlessly integrated into current traffic flows, operating schedules, and existing service infrastructure. Regrettably, comprehensive and accurate public data regarding these truck parking locations is hardly available.

## 3 Data Description

The data publication comprises a singular dataset containing eleven columns formatted in comma-separated values (.csv). In this format, columns are separated by commas, while decimal points are utilized as separators.

### 3.1 Truck parking categories

This dataset distinguishes four archetypal categories, resulting from predominant tag combinations of OSM and commercial software data for truck-specific Point of Interest (POI). This yields designated truck stops, general rest areas, fueling stations, and parking-only areas as well as specific combinations.

First, *Truck Stops* denote areas typically found along major highways, serving as comprehensive service areas offering refueling facilities, dining options, general and truck-specific service amenities, accommodations, and rest and shower facilities. These areas are usually accessible to the public.

Second, *Fueling* locations are predominantly service areas facilitating heavy-duty truck fueling, with limited additional services available. These areas are usually accessible to the public.

Third, *Rest Areas* - commonly situated along major highways - provide parking spaces along with potential general amenities like restaurants, shops, or basic restroom facilities. Fueling and truck-specific service amenities may not be provided. These areas typically allow public access.

Last, *Parking* locations encompass parking areas usually close to industrial zones, although detailed information might be incomplete. Access to these locations may be restricted but is usually public / semi-public.

### 3.2 Latitude and longitude information

The geospatial data in this dataset is formatted in WGS:84, providing a standardized reference system. We note that for each location, the final latitude and longitude information equals the centroid of the respective location cluster. This results from deploying the Mean-Shift algorithm to merge single nearby locations to one common larger location, thus condensing the dataset and reducing redundancies. See Fig. 2 (final, clustered) versus Fig. 4b (raw) as an examplary location hotspot.

### 3.3 Area information

The area data, denoted in square meters, was calculated by geometric information from OSM objects. The specified area represents the cumulative sum of the areas attributed to the respective cluster. When this geometric information is not accessible for the given location, the field remains empty. See the Data Collection and Clustering section for more details.

### 3.4 Truck parking confidence (TPC)

This TPC information comprises two attributes, namely *High* and *Medium*. Locations were labeled with a *High-TPC* when multiple information sources, a convergence of various tags, or the inherent category (such as Truck Stop) collectively imply a substantial probability of accessibility and suitability for trucks. A *Medium-TPC* was assigned to locations if a single information source or individual tags indicated a certain probability of truck accessibility and suitability. Conversely, locations received a *Low-TPC* rating and were subsequently excluded if discernible and reliable information was absent, incomplete, or where conflicting information prevailed.

### 3.5 Country information

This dataset encompasses the member countries of the European Union (EU-27), the European Free Trade Association (EFTA) countries, along with the United Kingdom (UK).

### 3.6 CORINE land cover (CLC)

This information represents the assigned land use/land cover defined by the CORINE Land Cover categories, distinguishing 44 thematic classes for the 2018 reference year. For example, this comprises thematic classes from artificial surfaces such as urban to industrial areas, agricultural areas from arable land to pastures, forests, semi-natural areas, or wetlands. This land use/land cover information is assigned to each location. See Fig. 6b and the Data Enhancement section for more details..

### 3.7 Minimal distance to the TEN-T road network

This information represents the minimal aerial distance to the TEN-T Core/Comprehensive road network, measured in kilometers. This field remains empty if the minimal aerial distance exceeds around 15 km to the TEN-T network. See Fig. 6a and the Data Enhancement section for more details.

### 3.8 Truck flow information

This information represents annual truck count data around the respective location. Either the truck counts from the nearest location or the maximum truck counts within a 5 km maximum radius. Aerial distances were used for calculation. This field remains empty if no truck count data is available within this maximum radius. Values are given in millions of trucks annually. See Fig. 6c and the Data Enhancement section for more details.

### 3.9 Overview

**Fig. 1:**
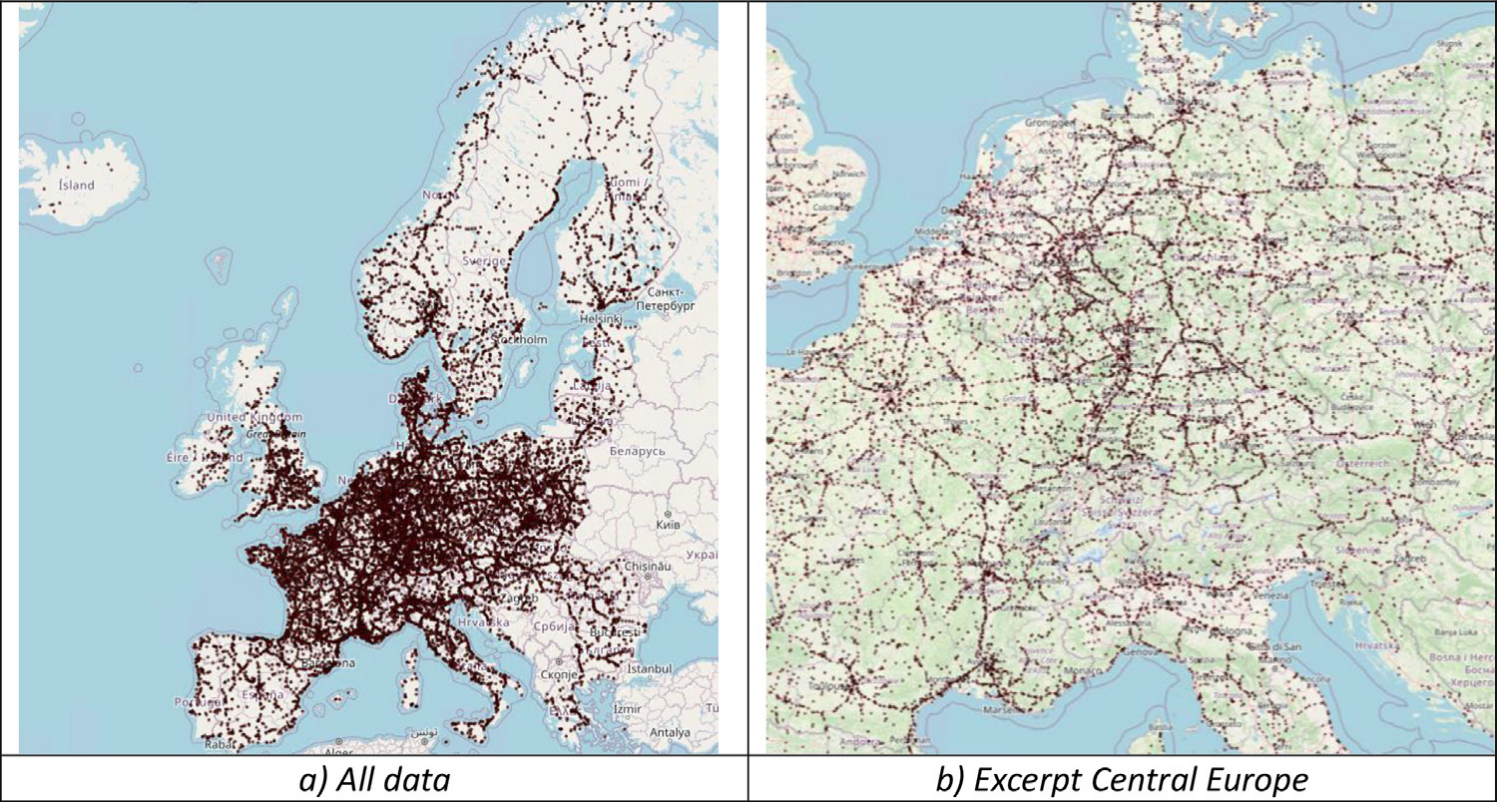
Data overview. Locations are visualized as small red circles. Country and regional boundaries are included. Own illustration based on Python Folium with map background from OSM.

**Fig. 2:**
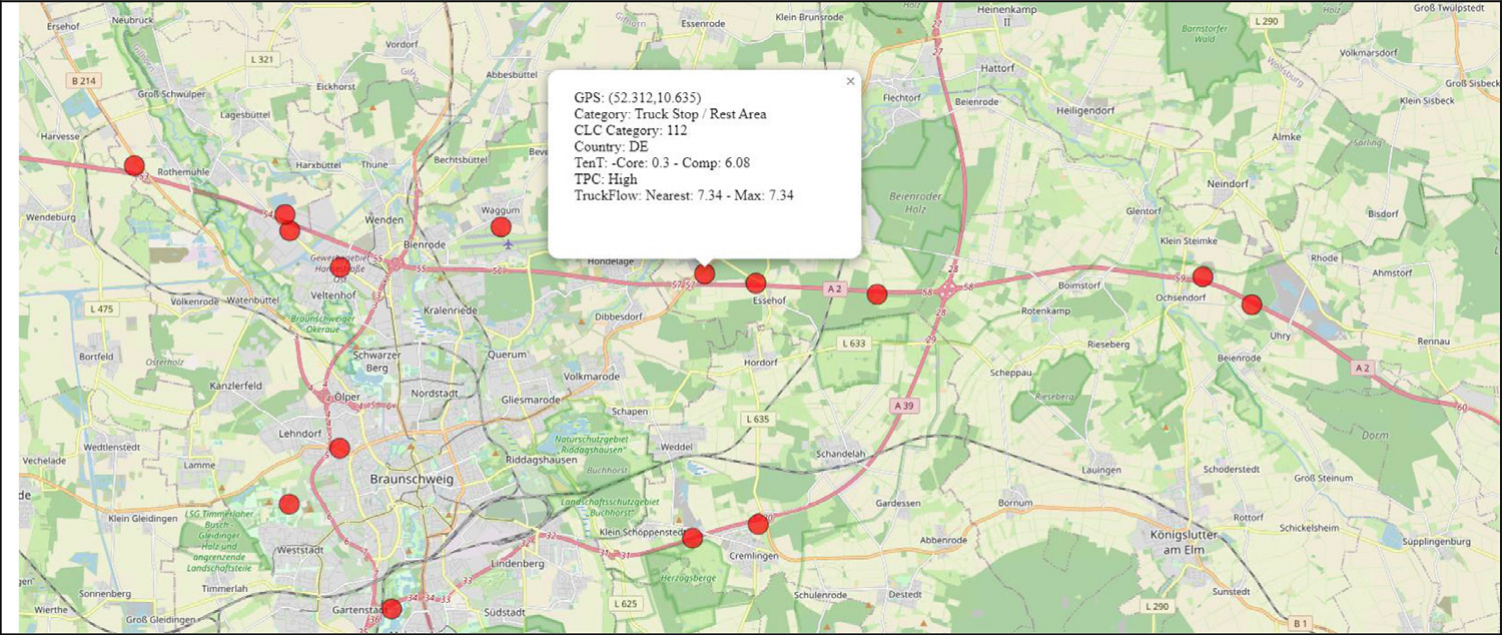
Zoom-level overview including associated information for the chosen location (#4712). Own illustration based on Python Folium with map background from OSM.

Fig. 1 visually illustrates the distribution of truck parking locations across Europe (a) and a more detailed excerpt from Central Europe (b). Meanwhile, Fig. 2 zooms in and presents the high resolution and precision of the data and related information about a location.

## 4 Experimental Design, Materials and Methods

**Fig. 3:**
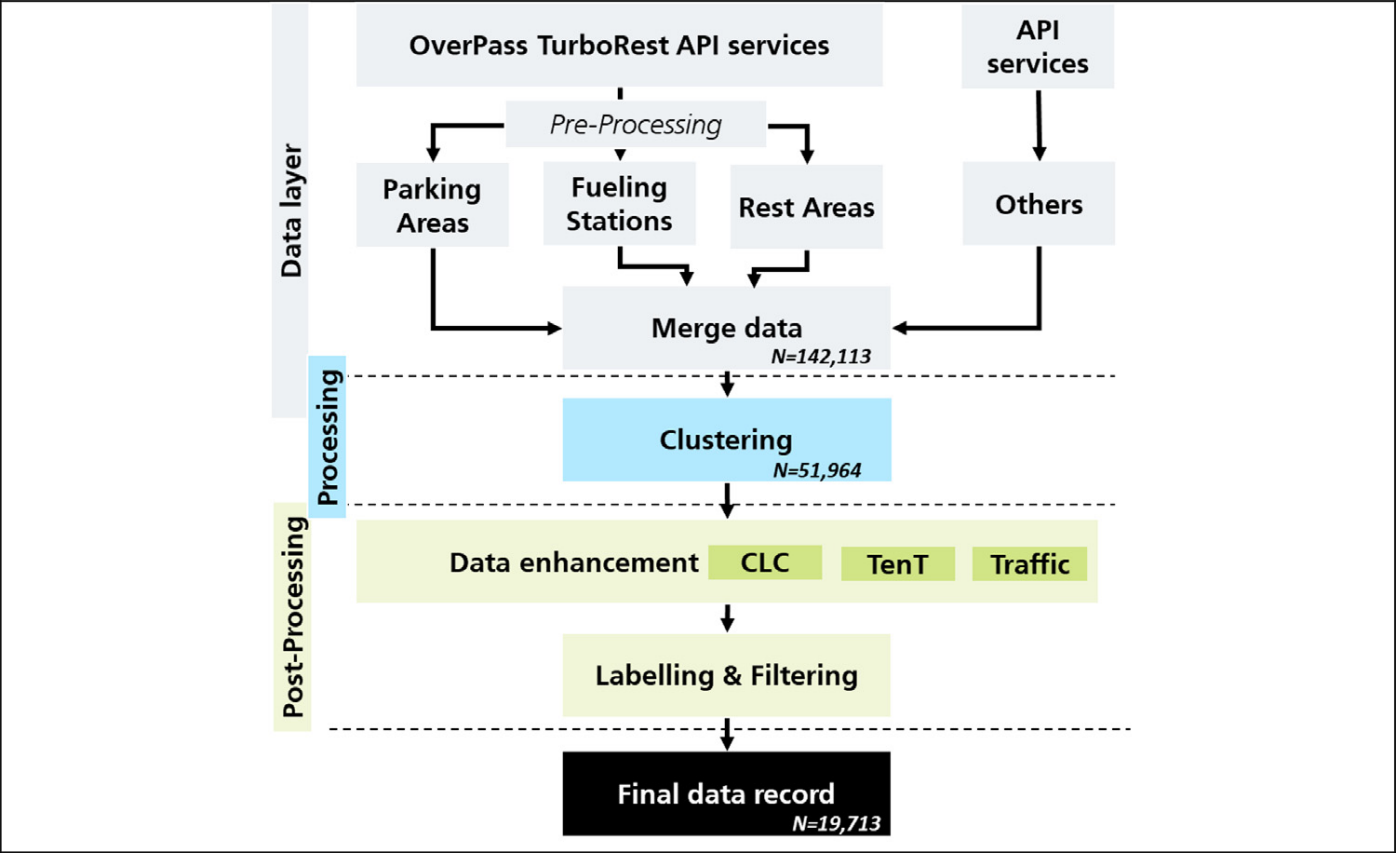
Data compilation process - overview. Sample size per stage is indicated. Own illustration.

Fig. 3 visualizes the structural layout and the methodological framework employed in compiling the data. It visualizes the sequence of pre- and post-processing filtering steps, illustrates the utilization of multiple data sources, and the clustering process to condense the dataset by merging nearby locations. We performed all calculations on a standard Lenovo notebook with i7–8565 U @1.8 GHz and 16 GB RAM. All code was written in Python 3.5.

### 4.1 Data collection

This paper used two primary data sources. All geospatial data was transformed or standardized to comply with the WGS:84 format, mitigating potential reference errors (Table 1).

**Table 1:** Specification table - overview of parameters and data types.

Variable	Type	Brief describtion
category	Categorical	Location category.
lat	Float	Latitude information. WGS-84 format.
lon	Float	Longitude information. WGS-84 format.
totalArea_m2	Integer	Estimated total area in square meters.
truckParkingConfidence	Categorical	Truck parking confidence label.
country	Categorical	Assigned country.
clc	Categorical	CORINE Land Cover inventory with 44 classes.
distance_TenTcore_km	Float	Calculated minimal distance to the TEN-T Core road network. In kilometers.
distance_TenTcomp_km	Float	Calculated minimal distance to the TEN-T Comprehensive road network. In kilometers.
truckFlowCount_nearest	Float	Number of trucks in the immediate vicinity, measured in millions per year.
truckFlowCount_max	Float	Maximum number of trucks in the vicinity, measured in millions per year.

First, we systematically collected OSM data [1] through countrywide queries utilizing the Overpass API [2]. The study assumed that public truck parking predominantly occurs at general parking areas, rest stops, or fueling stations. Noteworthy is OSM's incorporation of three fundamental types—nodes (1D), ways (2D), and relations (1D/2D). The geospatial information for each location encompassed geographical coordinates delineated by latitude and longitude pairs, encapsulating both the surrounding hull (ways, relations) and the corresponding centroid or center (nodes, ways, relations). Supplemental characterizations were provided through associated tags. These tags included general descriptors such as *capacity, access, name*, and *operator* alongside truck-specific tags like *hgv, capacity:hgv, hgv:lane, fuel:hgv_diesel*, and *hgv:lanes*. We highlight the fragmented nature of these OSM tags, meaning there is no standardized scheme - yet recommendations only - for tag assignment to particular POIs or enterable values/data formats per tag, complicating evaluations. Compared to the global OSM database, the extracted types per category should cover almost all relevant locations mapped in OSM. Table 2 provides a comprehensive summary of OSM tags and types.

**Table 2:** OSM summary of OSM tags and types. Own illustration.

OSM category	Query	Extracted Types	Estimated data completeness
Parking areas	*amenity=parking*	Nodes (1D), Ways (2D)	>99 %
Rest area	*highway=rest_area*	Nodes (1D), Ways (2D), Relations (1D/2D)	>99 %
Fueling station	*amenity=fuel*	Nodes (1D), Ways (2D)	>99 %

Second, commercial truck routing software served to validate OSM locations and supplement absent or incomplete information as well as missing locations. This process involved harnessing the geocoding capabilities offered by the PTV Developer API [3], TomTom Developer API [4], and HERE Developer API [5] and searching for the respective categories.

The initial data collection was followed by an extensive data cleaning to filter out non-truck relevant locations in the OSM extract. Specifically, the assessment of truck suitability and accessibility relied upon examining general and truck-specific OSM tags (see above). This also involved the assessment of spatial overlaps and proximity among various locations, both within categories and across categories. Plus, the enclosed area of each location (in square meters for 2D objects) was calculated using the *pyproj.Geod package* [14] and the *geometry_area_perimeter* function. Locations without indications of truck-relevance through OSM tags or nearby truck-relevant information suggested by the commercial truck routing software were excluded from further analysis, significantly reducing the dataset size for each category. Moreover, locations presumably limited to private use or designated for delivery (identified by OSM tags *access:private, access:delivery, hgv:private*, or *hgv:delivery*) were excluded. Table 3 shows the final overview per OSM category.

**Table 3:** Final overview per OSM category. Own illustration.

OSM category	Initial locations	Final data	Share of remaining data
Parking areas	*N* = 2661,731	*N* = 73,594	2.8 %
Rest area	*N* = 18,279	*N* = 8337	46 %
Fueling station	*N* = 131,374	*N* = 19,627	15 %

**Fig. 4:**
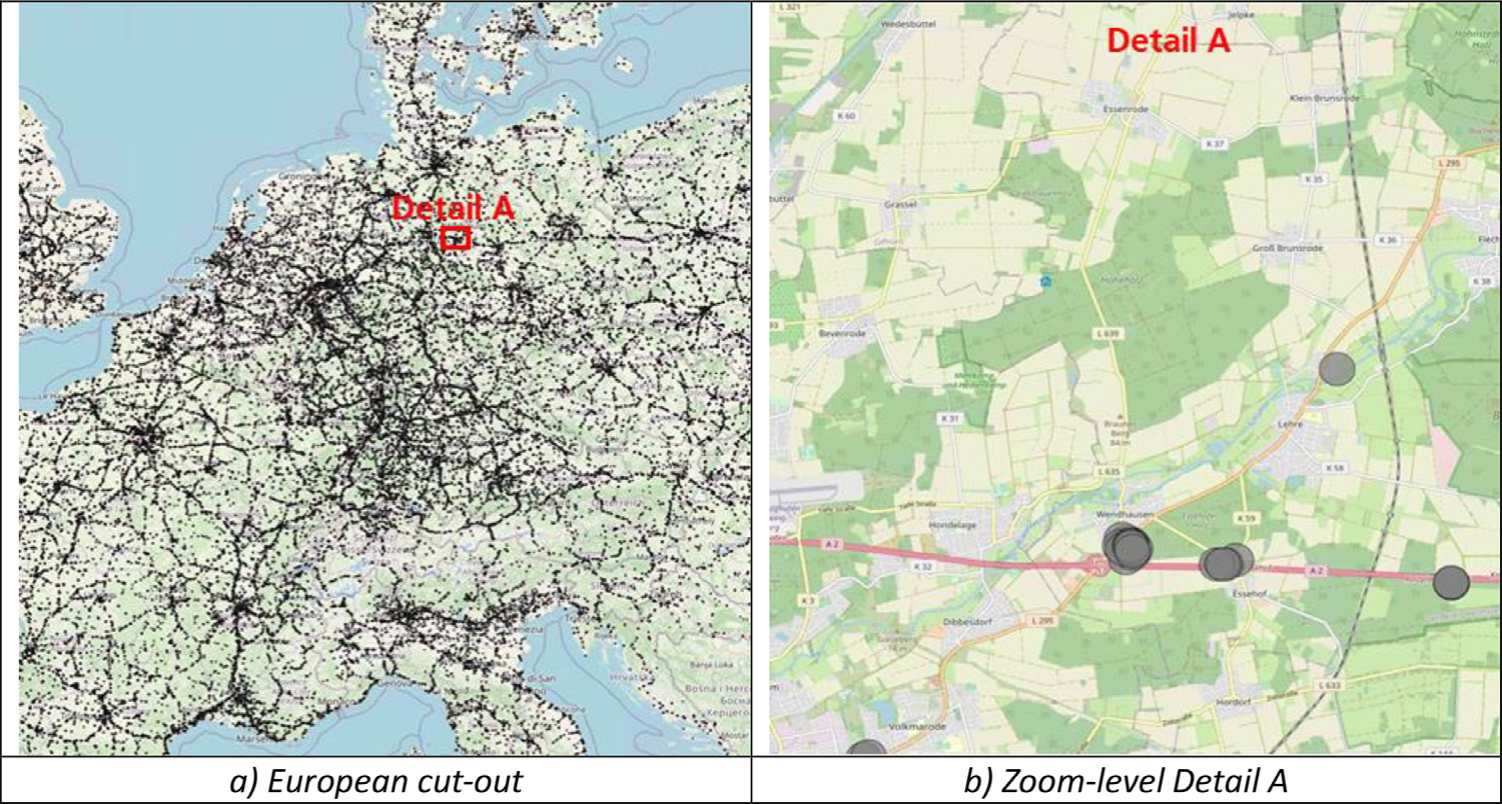
Overview of the merged dataset. Locations are visualized as small gray circles with country and regional boundaries. Own illustration based on Python Folium with map background from OSM.

Following the data cleaning, all locations were merged. Accordingly, additional locations sourced from the commercial truck routing software were added (*N* = 41,155). Consequently, the final dataset comprised a total of *N* = 142,113 locations. This result is visualized in Fig. 4, showing the overall perspective on the left and zooming in on Detail A on the right. This detailed view reveals intra-category and cross-category location hotspots, leading to redundancies and distortions.

### 4.2 Clustering

Further refinement was achieved through clustering to eliminate redundancy caused by location hotspots and to condense the dataset. The Mean-Shift clustering algorithm as implemented in the *Python sklearn.cluster module* [15] was employed for this purpose. This density-based algorithm offers flexibility in determining the number of centroids, exhibits robustness against outliers, and is not restricted by data shape or distribution [16]. The clustering process involved testing the distance to form clusters (bandwidth parameter) within 25 to 2100 m. The minimum number of points required to constitute a cluster was set to 1, and border points were included. An optimal distance (*d* = 250 m) was selected using the elbow method. This process yielded *N* = 53,699 locations, each represented by a cluster centroid that inherits and accumulates all information from its original locations.

**Fig. 5:**
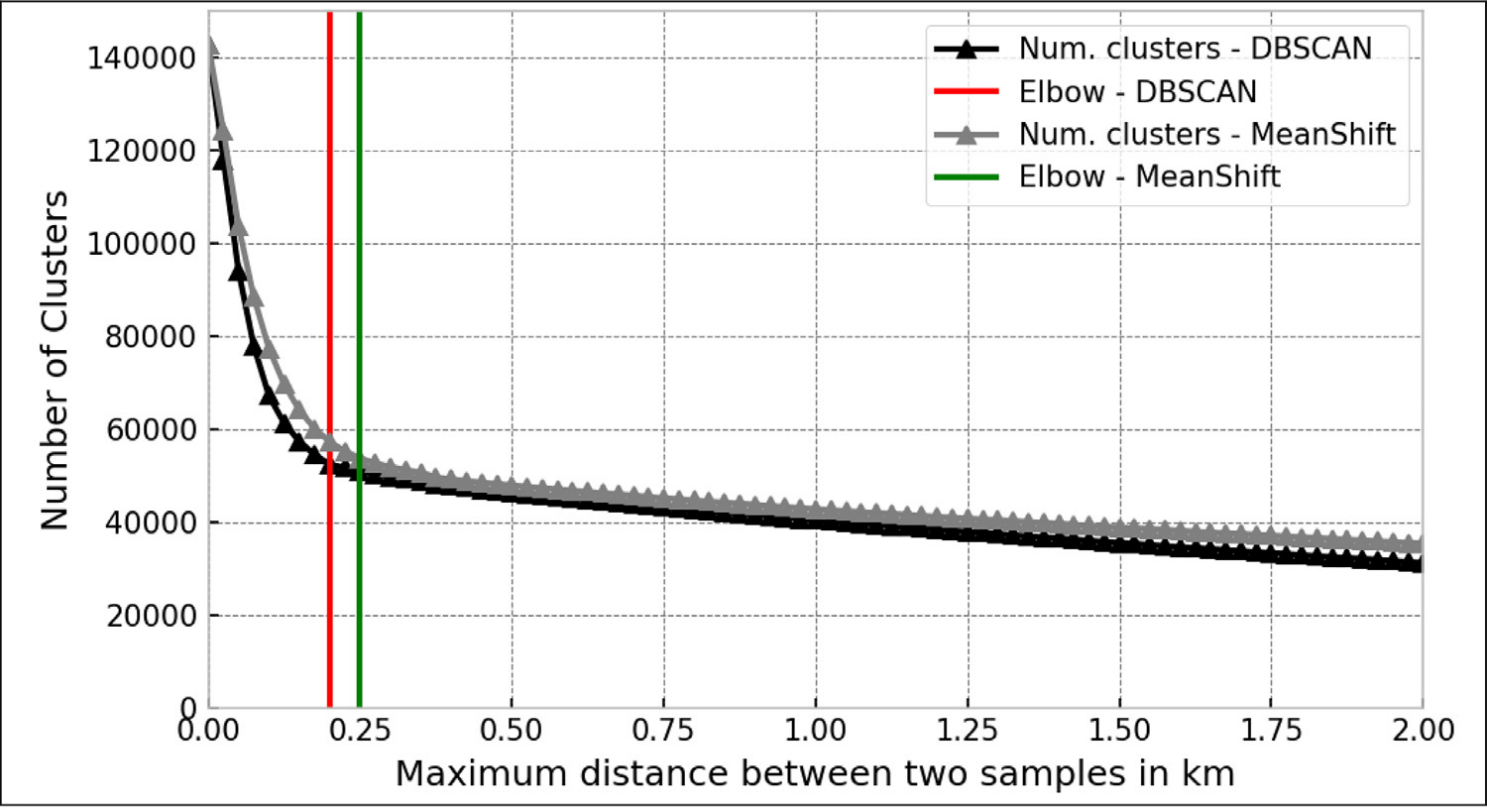
Comparison of clustering algorithms on the merged dataset (*N* = 142,113). Lines with triangles (black: DBSCAN, gray: Mean-Shift) show the effective number of clusters as functions of the clustering radius. Elbows are visualized as vertical lines (red: DBSCAN, green: Mean-Shift). Clustering radius were tested from 25 to 2100 m, with a step size of 25 m. (For interpretation of the references to color in this figure legend, the reader is referred to the web version of this article.)

These results were compared against the DBSCAN (Density-Based Spatial Clustering of Applications with Noise) algorithm for validation. Both algorithms are commonly used in geospatial data processing and clustering studies - DBSCAN: [17,18], Mean-Shift: [19,20]. As shown in Fig. 5, comparing both methods reveals similar outcomes, indicating an optimal distance between 200 m (DBSCAN, *N* = 52,364) and 250 m (Mean-Shift, *N* = 53,699).

Finally, all clustered locations (*N* = 53,699) were assigned to their respective countries, encompassing the EU-27, EFTA, and the UK. Locations falling outside the scope of interest, notably those in border regions, were excluded (*N* = 53,412). Additionally, the geographic proximity between all clustered locations has been calculated, and if locations were closer than 250 m, they have been re-merged. This additional processing resulted in *N* = 51,964 final clustered locations.

### 4.3 Data enhancement

The data enhancement process entailed the integration of three additional datasets to add value and information about the relevance of the locations concerning truck traffic. All additional and their geospatial data was standardized or transformed to the WGS:84 format to mitigate reference errors.

**Fig. 6:**
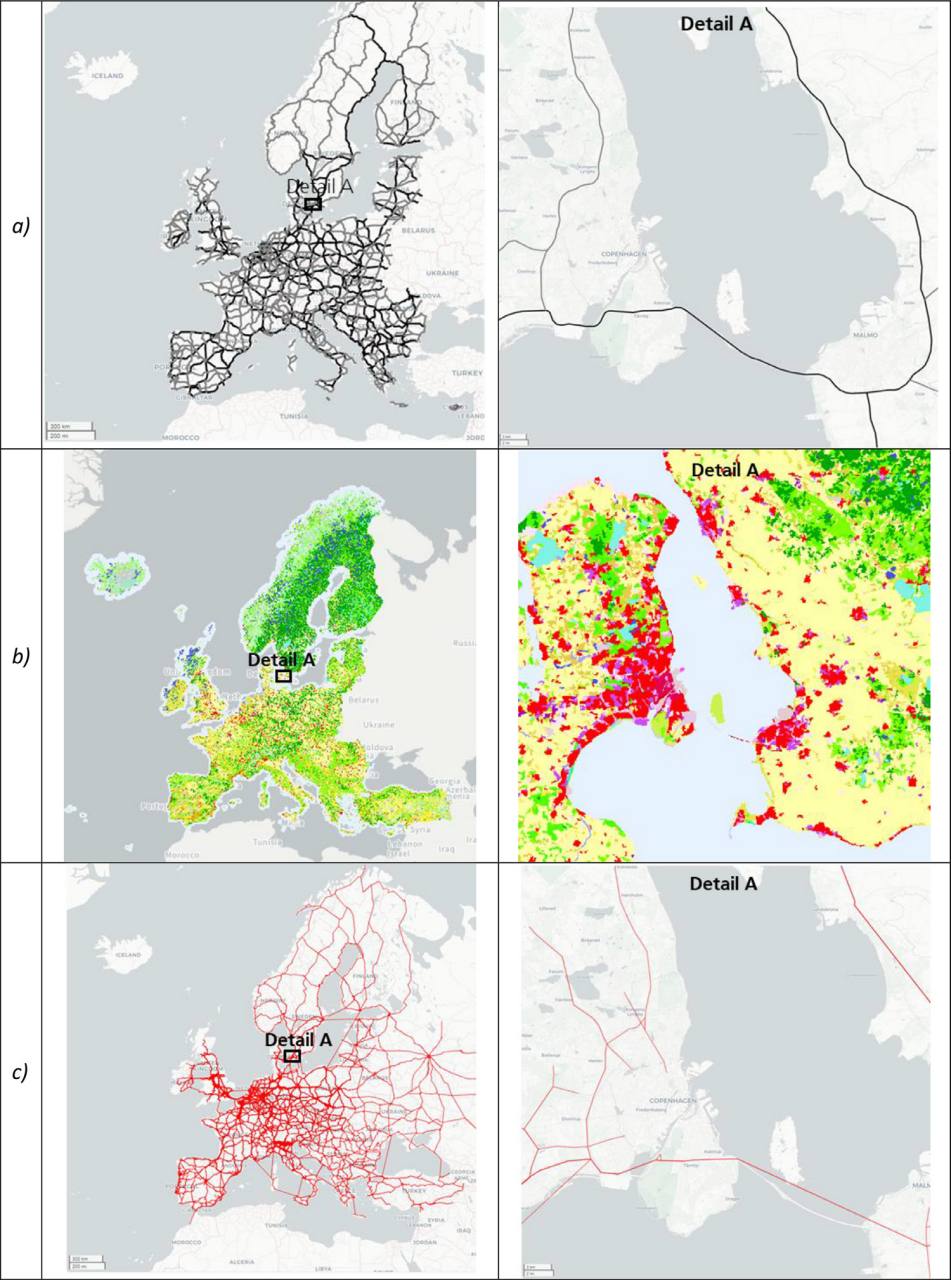
Data enhancement. a) Overview of the TEN-T road network and precision level. Black lines represent the Core network. Gray lines represent the Comprehensive network. Own illustration based on Python Folium with map background from CartoDB Positron. b) Overview of the CORINE Land Cover (CLC) data. Illustration taken from [7] with color-coded thematic classes c) Overview of the TTF network data and precision level. Red lines represent the network edges; linewidth is correlated to the annual truck traffic flow. Own illustration based on Python Folium with map background from CartoDB Positron. (For interpretation of the references to color in this figure legend, the reader is referred to the web version of this article.)

First, we added information about the proximity of locations compared to the main transport corridors. Accordingly, we calculated the minimal aerial distance of each location to the TEN-T road network. We distinguished between the Core and Comprehensive Networks, as defined in the data provided by the European Commission DG MOVE through the TENtec Information System 2022 [6]. Herein, road sections are defined as geometric LineString, representing a sequence of geospatial coordinate points. That being said, we determined the closest point on every LineString to every location and then computed the Haversine distance using the *pyproj.Geod package* [14]. We defined 15 km as cut-off and disregarded longer distances, as we focused on the main EU transport corridors. This already covers a considerable catchment or off-highway area, while major detours are hardly expected to be relevant in operations. This corresponds to a precision threshold of about 0.1 to 0.15 decimal degrees. Fig. 6a provides an overview of the TENtec data and their spatial resolution.

Second, the underlying land use/land cover per location was evaluated. We used the latest pan-European CORINE Land Cover (CLC) inventory, including 44 thematic classes for the 2018 reference year (100 m raster). For each location, we determined the specific raster or shape (2D shape, polygon) within which the corresponding geospatial coordinates were situated and assigned the pertinent CLC class. Fig. 6b shows an overview of the CLC-2018 data and their spatial resolution.

Third, supplementary information regarding nearby traffic flows was incorporated based on synthetic truck traffic flow (TTF) data provided by Speth et al. [8]. This network-based data represents the major European transport paths (frequently close to the TEN-T network) and assigns annual traffic flow information to each edge within the network. Each edge is defined by its origin and destination node and represents a geometric LineString. The further calculation follows analog to the TEN-T network. Distances surpassing a certain precision threshold (5 km) were disregarded. Fig. 6c showcases an overview of the TTF data and their spatial resolution.

### 4.4 Post-Processing

The post-processing phase encompasses two integral procedures referred to as labeling and filtering.

Labeling involves the creation of definitive category tags—*Truck Stop, Parking, Fueling, Rest Area*—based on the joined information per clustered location. Each location is classified as whether the information originated from only one source (OSM or commercial truck routing software) or a combination thereof.

Filtering comprises two distinct steps and commences by implementing default TPC (truck parking confidence) settings derived from these labels and the information origin: *Truck Stops* are marked with *High-TPC*, Rest Areas acquire *High-* or *Medium-TPC*, while Fueling and Parking locations receive *Medium-* or *Low-TPC*. Finally, locations are evaluated based on their default label, available area information, proximity to the TEN-T network, CLC information, and area access information to confirm or update the default TPC label. The final dataset exclusively retains locations possessing *Medium-* or *High-TPC* (*N* = 19,713). Table 4 provides a conclusive overview.

**Table 4:** Final dataset. Overview of categories (rows) and truck parking confidence labels (columns).

Category	High	Medium	Low
Fueling	329	6064	7742
Fueling / Truck Stop	1260		0
Parking	464	4204	22,663
Parking / Rest Area	525		0
Rest Area	4120	2351	534
Truck Stop / Rest Area	275	121	0
Total dataset	6973	12,740	0
Total	19,713		–

## Limitations

While we have combined multiple data sources to enhance accuracy and broaden coverage in our truck stop location dataset, we cannot ensure absolute data completeness. We highlight that coverage varies among countries. Moreover, occasional information gaps and inconsistencies exist in the OSM data and commercial truck routing software, where particularly non-standardized OSM data complicate evaluations. To mitigate these challenges, we have implemented a series of filtering mechanisms and established truck parking confidence levels. We have also defined location categories based on the most reliable information available. This approach should ensure the dataset's credibility, utility, and certain accuracy.

The area calculation using the enclosed area defined by OSM polygons exhibits limitations, occasionally encompassing roads, grasslands, and other service areas within its boundary. Moreover, only certain areas might be designated as truck parking zones. Hence, not all of the estimated area guarantees truck accessibility or parking.

Please note that final latitude and longitude information may represent the centroid of the respective location cluster that originated from mean-shift clustering and filtering. Thus, the final coordinates may not perfectly align with exact geographical coordinates of single locations. We propose considering and utilizing these locations as candidates for conducting in-depth local analyses concerning ambient conditions and truck parking suitability.

Last, we highlight that future research may integrate additional information on safe and secure truck parking areas (SSTPAs) in Europe, whose development and extension along the TEN-T network is highly prioritized and promoted by the European Commission. Those parking areas are upgraded and certified to provide proper security for drivers and cargo and improve driver comfort. This SSTPA label may substantially boost the attractiveness of locations for deploying alternative recharging or refueling infrastructure.

## Ethics Statement

The authors have read and follow the ethical requirements for publication in Data in Brief and confirming that the current work does not involve human subjects, animal experiments, or any data collected from social media platforms.

## Declaration of AI and AI-Assisted Technologies in the Writing Process

During the preparation of this work the authors used “Grammarly” for English editing. After using this tool, the authors reviewed and edited the content as needed and take full responsibility for the content of the publication.

## CRediT authorship contribution statement

**Steffen Link:** Conceptualization, Methodology, Software, Formal analysis, Data curation, Writing – original draft. **Patrick Plötz:** Conceptualization, Writing – review & editing, Supervision, Project administration, Funding acquisition.

## Data Availability

European Truck Parking Locations (Original data) (Zenodo).

## References

[1] OpenStreetMap contributors (2017). https://planet.osm.org.

[2] Olbricht R. (2023). https://github.com/drolbr/Overpass-API.

[3] PTV Developer (2023). https://developer.myptv.com/en.

[4] TomTom Developer (2023). https://developer.tomtom.com/.

[5] Here Developer (2023). https://developer.here.com/.

[6] European Commission (2022). https://ec.europa.eu/transport/infrastructure/tentec/tentec-portal.

[7] European Environment Agency (2019).

[8] Speth D., Sauter V., Plötz P., Signer T. (2022). Synthetic European road freight transport flow data. Data Br..

[9] Creutzig F., Jochem P., Edelenbosch O.Y., Mattauch L., van Vuuren D.P., McCollum D., Minx J. (2015). Energy and environment. Transport: a roadblock to climate change mitigation?. Science.

[10] Jaramillo P., Kahn Ribeiro S., Newman N., Dhar S., Diemuodeke O., Kajino T., Lee D.S., Nugroho S.B., Ou X., Strømman A.H., Whitehead J. (2023). Climate Change 2022: Mitigation of Climate Change: Contribution of Working Group III to the Sixth Assessment Report of the Intergovernmental Panel on Climate Change.

[11] Anderhofstadt B., Spinler S. (2019). Factors affecting the purchasing decision and operation of alternative fuel-powered heavy-duty trucks in Germany – A Delphi study. Transport. Res. Part D.

[12] Bae Y., Mitra S.K., Rindt C.R., Ritchie S.G. (2022). Factors influencing alternative fuel adoption decisions in heavy-duty vehicle fleets. Transport. Res. Part D.

[13] Sugihara C., Hardman S., Kurani K. (2023). Social, technological, and economic barriers to heavy-duty truck electrification. Res. Transport. Bus. Manag..

[14] Whitaker J. (2023). https://pypi.org/project/pyproj/.

[15] Pedregosa Fabian, Varoquaux Gaël, Gramfort Alexandre, Michel Vincent, Thirion Bertrand, Grisel Olivier, Blondel Mathieu, Prettenhofer Peter, Weiss Ron, Dubourg Vincent, Vanderplas Jake, Passos Alexandre, Cournapeau David, Brucher Matthieu, Perrot Matthieu, Duchesnay Édouard (2011). Scikit-learn: machine learning in python. J. Mach. Learn. Res..

[16] Cheng Y. (1995). Mean shift, mode seeking, and clustering. IEEE Trans. Pattern Anal. Machine Intell..

[17] Garcia J.C., Avendaño A., Vaca C., Rocha Á., Adeli H., Reis L.P., Costanzo S. (2018). Trends and Advances in Information Systems and Technologies: Volume 1.

[18] Plötz P., Speth D. (2021). https://www.isi.fraunhofer.de/content/dam/isi/dokumente/cce/2021/ACEA_truckstop_report_update.pdf.

[19] Salguero F., Prat F., Moreno F., Romero S. (2011). Mean-Shift: a non-parametric algorithm for the segmentation of anomalies in geophysical images obtained from magnetic prospection data. Archaeometry..

[20] Magiya J. (2019). https://levelup.gitconnected.com/clustering-gps-co-ordinates-forming-regions-4f50caa7e4a1.

